# Laser confers less embryo exposure than acid tyrode for embryo biopsy in preimplantation genetic diagnosis cycles: a randomized study

**DOI:** 10.1186/1477-7827-9-58

**Published:** 2011-04-28

**Authors:** Selmo Geber, Renata Bossi, Cintia B Lisboa, Marcelo Valle, Marcos Sampaio

**Affiliations:** 1 ORIGEN, Centro de Medicina Reprodutiva, Av. Contorno 7747, Lourdes, CEP 30110-120, Belo Horizonte, Minas Gerais, Brazil

## Abstract

We compared two methods of zona pellucida drilling. 213 embryos were biopsied with acid Tyrode. Each biopsy took 3 minutes and the entire procedure ~29 minutes. 5% of blastomeres lysed, 49% of embryos became blastocyst and 36% of patients became pregnant. 229 embryos were biopsied with laser. Each biopsy took 30 seconds and the entire procedure ~7 minutes. 2.5% of blastomeres lysed, 50.6% of embryos became blastocyst and 47% of patients became pregnant. We can conclude that laser can be used for embryo biopsy. Reduction of embryo exposure and of removed blastomeres is associated with increased blastocysts available for transfer and a better clinical outcome.

## Background

With the development of preimplantation genetic diagnosis (PGD) technique for the detection of inherited diseases [1] biopsies of one or two cells collected from embryos in the pre-implantation stage post IVF [2] have been performed to avoid the transfer of affected embryos. To remove the blastomeres, the zona pellucida (ZP) must be opened. Various approaches have been used that apply different micromanipulation procedures for ZP drilling [3-12].

Initially, these procedures were performed to enhance fertilization rates [4] and for assisted hatching [5], in which the drilling of the ZP was performed either chemically or mechanically. For PGD, an acidified Tyrode's solution has been used to open the ZP, without causing adverse effects on embryo development [6,8,12]. Alternatively, partial dissection of ZP using a fine needle to penetrate the zona and a holding pipette to create the hole has been done [5].

More recently, non-contact lasers within the infrared range were used for drilling the ZP in mice, with no indication of negative effects on embryo development [7,13]. Also, laser assisted hatching has been applied in human embryos [10,11], producing results similar to those obtained with other methods [3,9].

Although embryo biopsy for PGD is a well established method used worldwide it is not known whether the method of choice for ZP drilling affects further embryonic development or pregnancy rates. Also, many centers seem to be reluctant in using the laser drilling technique for PGD. The incorporation itself of the PGD in the routine of a fertility lab already imposes some changes and requires that new protocols and training of specialized staff are established. This may explain why only a limited number of papers have been published on the use of laser for ZP drilling [14,15].

In this study we compared the two different methods for ZP drilling, namely laser and chemical, in human embryo biopsies submitted to PGD. For each method we analyzed the number of blastomeres removed from each embryo, the time required for embryo biopsy, how many times the incubator had to be opened, blastocyte formation and number of blastocytes transferred, and pregnancy rates.

## Methods

### Patients

A total of 74 couples undergoing IVF/ICSI for PGD due to a risk of transmitting genetic disorders were included in the study. The 74 couples underwent 74 IVF/ICSI cycles at ORIGEN - Centro de Medicina Reprodutiva - Belo Horizonte - Brazil. This study was approved by our local ethics committee and patients signed informed consent. Indications for PGD were chromosomal abnormalities (28-37.8%), risk of aneuplody (32-43.2%) and monogenic disease (14-18.9%). Couples were randomly allocated in two groups according to the method of ZP drilling. Thus, 36 couples were submitted to chemical drilling with acid Tyrode's solution (ATZD) and, and 38 couples to laser drilling (LZD).

All patients were submitted to complete infertility evaluation and only women who had serum follicle stimulating hormone (FSH) levels <15 pg/ml on day 3 were included in the study. Semen evaluation was performed according to the recommendations of the World Health Organization [16]. Intracytoplasmic sperm injection (ICSI) was performed in all cases. Pregnancy rate was defined as gestational sac with heart beat seen with ultrasound per embryo transfer.

The randomization of this study was performed in accordance do the IVF-PGD cycles, but not the embryos, to ensure that the observed result occurred to embryos submitted to each specific technique.

### Ovulation Induction

All patients underwent the same protocols for ovulation induction using the same hormones and criteria for dose tailoring. Treatment started with administration of gonadotrophin releasing hormone analogues (Triptorelyn-Gonapeptyl-Ferring-Brazil) for suppression of the pituitary function. To confirm down-regulation, serum estradiol (E_2_) levels and vaginal ultrasound were performed ~10 days later. If the E_2 _concentration was <30 pg/mL and the ultrasound showed an endometrial thickness <3 mm, patients were considered ready to start ovulation induction. If patients were not ready, serum E_2 _and ultrasound were repeated every other day until suppression was achieved [17].

After confirmation, patients were superovulated with recombinant FSH (GonalF-Serono-Brazil). The starting dose of rFSH was defined according to patient's age and was tailored according to the ovarian response measured by E_2 _levels and follicular growth monitored by ultrasound (Tosbee-Toshiba-Japan). Recombinant hCG (rhCG-Ovidrel-Serono-Brazil) was given when at least 3 follicles reached a mean size of 17 mm with concordant E_2 _levels (~200 pg/mL) [18].

### ICSI procedure

Oocyte retrieval was performed ~34 hours after rhCG injection by vaginal ultrasound guided aspiration. Oocytes were inseminated 4 hours later (day 0) by ICSI, after mechanical denudation with 80 IU/ml of hyaluronidase. Semen preparation was performed with the swim-up technique. Micromanipulation was carried out on a heated stage of a Nikon Diaphot inverted microscope at X400 magnification (Nikon-Japan) adapted with a pair of hydraulic micromanipulators and a motor-driven coarse control (Narishige-Japan). A single spermatozoon was initially aspirated from a drop of HEPES-buffered Earle's balanced salt solution (Sigma-USA), containing 10% of polyvinylpirolidone (PVP-Irvine-USA) and injected through the zona pellucida into the cytoplasm [19].

Approximately 17 to 19 hours later (day 1) the oocytes were checked for normal fertilization by the presence of two pronuclei. The embryos were cultured in 20 μl droplets of Earle's balanced salt solution (Sigma) with 10% synthetic serum substitutive at 37°C in a Petri dish (Falcon-BD-USA) under mineral oil (Sigma) and a gas phase of 5% CO2. Day 3 embryos were transferred to 20 μl droplets of S2 (Scandinavian IVF-Sweden) until day 5. After diagnosis, a maximum of 3 best quality blastocysts were transferred [20].

The luteal phase was supported with vaginal progesterone (Crinone8%-Serono-Brazil) [21]. Serum hCG levels were measured 12 days after embryo transfer. Confirmation of pregnancy was made by ultrasonography two and four weeks later.

### Chemical and laser cleavage stage embryo biopsies

For both procedures, normally fertilized embryos grade I/II, which had reached the 5- to 10-cell stage on day 3 were transferred into drops of Ca/Mg-free medium (sigma) in a Petri dish under mineral oil. Micromanipulation was done on a heated stage of an inverted microscope adapted with a pair of hydraulic micromanipulators. Each embryo was immobilized by suction on a holding pipette held in one micromanipulator. For the chemical biopsy, the second micromanipulator with a double holder controlled a drilling pipette (internal diameter*10*m) containing acid Tyrode's solution (pH 2.2) and a sampling pipette (internal diameter*30*m) containing medium. The drilling pipette was placed in close contact with the ZP and a hole was made with a controlled stream of acid Tyrode. This pipette was removed, and the sampling pipette pushed through the hole.

For laser, each embryo was immobilized by suction on a holding pipette held in the micromanipulator and after correct positioning of the ZP using the target generator, a hole was made by two (exceptionally three) pulses of 10-12 ms of laser (Octax Laser-Germany).

For both procedures, after the zona was penetrated, a sampling pipette was pushed through the hole and one cell at the equivalent of the 8-cell stage was removed by gentle suction. In all cases, an interphase nucleus was observed in the isolated blastomeres [22]. If lysis of the removed blastomere was observed, a second blastomere was then removed.

### Statistics

The χ^2^-test was applied to compare rates of blastocyst formation, pregnancy and lysis of blastomeres. Student t-test was performed to compare the number of transferred embryos. The Mann-Whitney test was used to calculate differences in time taken for biopsy and the number of times the incubators were opened. Differences were considered significant when p < 0.05.

## Results and discussion

The mean age (±SD) of the 36 women submitted to PGD using ATZD was 36.6 ± 1.4 years (range 30-41) and for the 38 patients who had LZD was 36.4 ± 1.4 (range 29-41). A total of 213 normally fertilized grade I/II embryos with 5-10 cells were biopsied using ATZD, with an average of 5.9 embryos per patient (range 2-8). In the group of patients that had LZD, 229 normally fertilized grade I/II embryos with 5-10 cells were biopsied, with an average of 6 embryos per patient (range 2-10). Initially one blastomere was removed from each embryo and 11 blastomeres (4.9%) lysed after ATZD and 6 blastomeres (2.5%) lysed after LZD (p = 0.2) (Table 1). The average number of blastomeres biopsied per embryo was 1.05 and 1.03, for ATZD group and LZD group, respectively.

**Table 1:** Human embryo biopsy characteristics after zona drilling using acid Tyrode or laser for PGD

	ATZD	LZD	p
PGD Cycles	36	38	
Biopsied Embryos	213	229	
Biopsied Embryos/patient ^a^	5.9	6	0.3
Blastomeres removed	224	235	
Blastomeres lysed	11 (5%)	6 (2.5%)	0.2

Note: ATZD = acid tyrode zona drilling. LZD = laser zona drilling.

^a^mean

The time needed for embryo biopsy when using ATZD was 180 ± 7 seconds per embryo and 30 ± 3 seconds per embryo if LZD was performed (p < 0.001). The overall time taken to biopsy all embryos ranged from 5 to 45 minutes (mean = 29 ± 7 min) when ATZD was performed and ranged from 2 to 15 minutes (mean = 7.6 ± 4 min) when LZD was performed (p < 0.001) (Table 2). We also evaluated how many times the incubators were opened to take the embryos from the incubator to the microscope and back to the incubator. In the group of patients whose embryos had ATZD the number of times ranged from 1 to 8 (mean = 5.1 ± 2.8), and in the group of patients whose embryos had LZD, this number ranged from 1 to 3 (mean = 1.6 ± 1.4) (p < 0.001).

**Table 2:** Laboratorial and clinical results of human embryo biopsy using acid Tyrode or laser for PGD

	ATZD	LZD	p
Time per embryo biopsy ^a^^b^	180	30	<0.001
Total time for procedure ^a^^c^	29	7.6	<0.001
Opening of incubator ^a^	5.1	1.6	<0.001
Blastocyst	105 (49.2%)	116 (50.6%)	0.8
Transferred blastocysts ^a^	1.9	2.1	0.3
Pregnancy	13 (36%)	18 (47%)	0.3

Note: ATZD = acid tyrode zona drilling. LZD = laser zona drilling.

^a^mean

^b^seconds

^c^minutes

The rate of blastocyst formation in the group submitted to ATZD was 49.2%, whereas in the group submitted to LZD was 50.6% (p = 0.8). The number of normal blastocysts transferred ranged from 1 to 3 and 70 were transferred in the ATZD group (mean = 1.9 ± 0.4 per transfer) and 82 in the LZD group (mean = 2.1 ± 0.5 per transfer) (p = 0.3). The overall pregnancy rate was 41.9% as 13 patients achieved pregnancy in the ATZD group (36%) and 18 in the LZD group (47%) (p = 0.3) (Table 2). A total of 5 miscarriages were observed, 2 in the ATZD (15.4%) and 3 in the LZD (16.6%).

The technique of embryo biopsy is a well-established procedure for PGD and has been used clinically for 20 years [1]. But, similar to other procedures that take place in the ART lab, small changes related to the microenvironment, the way temperatures of embryo cultures are recovered and maintained, and how often embryos are manipulated, to mention a few, may affect the successful development of good embryos [23]. Thus, small and apparently undetectable changes may explain the differences in pregnancy rates obtained by fertility clinics worldwide.

In our study, both groups were similar for patients' age and number of good quality embryos that were biopsied. The number of embryos successfully biopsied was similar in both groups and all embryos had at least one cell analyzed. The rate of blastocyst formation after biopsy was similar in both groups. The rates of blastocyst formation after embryo biopsy observed are similar or higher than the previously described either using ATZD [6,8] or LZD [24], or the described in studies in which both techniques were compared [14,25]. Joris et al. (2003) observed similar embryo development with either technique, although they did not follow embryo development until blastocyst stage.

Our study shows that LZD resulted in more intact blastomeres, as only 2.5% lysed in this group as compared to 5% in ATZD. This difference, although not statistically significant, may be explained by the direct exposition of the biopsied cell to acid Tyrode solution even though a controlled stream of acid is applied during drilling. Moreover, the lysis observed in the blastomeres after LZD occurred during manipulation of the blastomere for fixation, and not during drilling and aspiration. Reducing the number of blastomeres that are removed for biopsies is crucial, as it has been shown that the clinical outcome of 1-cell biopsy is significantly better than that of 2-cell biopsy [24]. The observed difference in lysis rates is similar to those described by others [14,15].

Embryo biopsy performed with LZD took significantly less time than that with ATZD. This difference was observed when we compared the time taken per embryo (30 × 180 sec) and relies mainly on the time taken to open zona pellucida. This is explained by the fact that with laser, only two pulses of 10 ms were necessary whereas for the acid solution the stream must be applied slowly in order to avoid damages to the cell membrane. As LZD biopsy is very fast, 3 embryos could be displaced in each petri dish at each time for the procedure. For ATZD biopsy, only one embryo was put in the petri dish at each time. Therefore, LZD also took significantly less time for the entire procedure (7 vs. 29 min). This led to less embryo and blastomere exposure. This difference in the duration of the procedure has not been previously evaluated and may be related to an increase in pregnancy rates.

We also compared how many times the incubator was opened according to each procedure. When using the laser technique, the incubator was opened significantly fewer times (1.6 vs. 5.1). This occurred because the number of embryos placed in the petri dish for each procedure was different. This may have an impact on embryo development, as the time-lapse for temperature recovery after the 5-sec opening of the door has been described to be approximately 30 min [23].

It is well known that temperature maintenance in the IVF lab is crucial for oocyte integrity and embryo development. Thus, a technique that affects the temperature of cell cultures less should be favored. Also, the fact that the laser creates a hole of known dimensions through the ZP, while the size of the hole created by the acid method is unknown, makes LZD less operator-dependable and better controllable, which ultimately may improve outcomes. In our study any potential benefits of LZD over ATZD that could lead to higher pregnancy rates could not observed, most likely due to the limited number of patients. The miscarriage rate however was similar in both groups.

## Conclusions

Our results show that the time required for embryo biopsy was significantly shorter when using laser; the number of times the incubator was opened was also shorter when using the laser. This led to less embryo and blastomere exposure, reducing the need of a second blastomere removal. Although we did not observe an increased in pregnancy rates, it is possible that a study with a larger group will reveal that the shorter time required with laser may lead to a increase in pregnancy rates.

## Competing interests

The authors declare that they have no competing interests.

## Authors' contributions

SG: study conception, execution, data analysis and interpretation, drafting of the manuscript. RB: execution, data collection, drafting of the manuscript. CBL: execution, data collection, drafting of the manuscript. MV: Data analysis and interpretation, critical disussion. MS: study conception, data analysis and interpretation, critical discussion. All authors read and approved the final manuscript.
